# Therapeutic Anti-Tumor Vaccines: From Tumor Inhibition to Enhancement

**DOI:** 10.4137/cmo.s538

**Published:** 2008-04-29

**Authors:** Paula Chiarella, Verónica Reffo, Juan Bruzzo, Oscar D. Bustuoabad, Raúl A. Ruggiero

**Affiliations:** División Medicina Experimental (ILEX-CONICET), Academia Nacional de Medicina de Buenos Aires, Pacheco de Melo 3081, 1425, Buenos Aires, Argentina

**Keywords:** tumor, immunotherapy, immunostimulation

## Abstract

Numerous immunization trials have proved successful in preventing the growth of experimental animal tumors and human hepatocarcinomas induced by hepatitis B virus. These results have prompted researchers and physicians to use vaccines in a therapeutic mode but the results have, in general, been disappointing even when strongly immunogenic murine tumors were concerned. Data presented herein suggest that immunotherapy induced by a single dose of a dendritic cell-based vaccine against a murine established tumor or against residual tumor cells after debulking the primary tumor, can render not only inhibitory or null but also stimulatory effects on tumor growth. These different effects might be dependent on where the system is located in the immune response curve that relates the quantity of the immune response to the quantity of target tumor cells. We suggest that high ratios render tumor inhibition, medium and very low ratios render null effects and low ratios—between medium and very low ones—render tumor stimulation. Since the magnitude of these ratios would depend on the antigenic profile of the tumor, the immunogenic strength of the vaccine used and the immunological state of the host, studies aimed to determine the magnitude of these variables in each particular case, seem to be necessary as a pre-condition to design rational immunotherapeutic approaches to cancer. In contrast, if these studies are neglected, the worst thing that an immunotherapist could face is not merely a null effect but enhancement of tumor growth.

## Introduction

The aim of all therapeutic approaches for cancer is the eradication of the tumor cells without causing major damage to the organism.

Conventional therapies, such as surgery, radiation and chemotherapy are limited by their local effects or by their lack of tumor-specificity and their toxic consequences. On the other hand, an immunological therapy would, at least theoretically, circumvent such limitations, because it could discriminate between the cells of the tumor and their normal cell counterparts wherever they are in the body.

For the last 50 years, numerous immunization trials aimed to prevent the growth of some experimental animal tumors and human hepatocarcinomas induced by hepatitis B virus, proved to be successful (Prehn and Main, 1957; Klein et al. 1960; O’Brien et al. 2004; Sioud, 2007). These promising results prompted researchers and physicians to use immunological schedules in a therapeutic mode. However, to date, most of the attempts to cause an immunologically—mediated regression of human established tumors have been unsuccessful (Finn, 2003; Cranmer et al. 2004; Vieweq et al. 2007).

Failures of anti-tumor immunotherapeutic schedules observed in clinical settings have, usually, been attributed to the putatively weak immunogenicity of many common human cancers on the basis that spontaneous tumors of mice and rats display, in general, lower immunogenicity than experimental ones, induced by massive doses of chemicals and viruses (Hewitt et al. 1976; Middle and Embleton, 1981; Ruggiero et al. 1985; Franco et al. 1996).

On this basis, attempts have been made to develop more efficient vaccines based on tumor cells made more immunogenic by the addition of adjuvants or by transfection with genes encoding co-stimulatory molecules or cytokines that enhance T-cell immune responses or by pulsing dendritic cells with tumor-cell extracts or tumor antigens (Janeway et al. 2001).

Presumably, the implicit assumption for these attempts is the belief that, as far as immunotherapy against established tumors is concerned, treatment of strongly immunogenic tumors is much easier than treatment of weakly immunogenic or non-immunogenic ones.

But, is this belief absolutely true?

Assuming that animals can be immunized against the growth of a tumor implant by using a given vaccination strategy (*preventive vaccination*), does it mean that the same strategy will be efficient for treating the same tumor once it becomes established (*therapeutic vaccination*)?

If not, is a null effect on tumor growth the worst possible outcome resulting for the application of immunological therapies, or upon certain circumstances, can growth of established tumors even be accelerated?

In this paper, we have attempted to address these questions by using a strongly immunogenic murine fibrosarcoma (MC-C) as tumor test, and dendritic cells loaded with tumor-lysate (mDC) as vaccination strategy. We evaluated MC-C tumor growth in mice receiving a single dose of mDC *prior* (preventive vaccination) or at different times *after* (therapeutic vaccination) tumor inoculation.

## Materials and Methods

### Animals

BALB/c mice of both sexes, 2–4 months old were used throughout. They were raised in our own colony and maintained on Cooperation pellets (San Nicolás, Buenos Aires, Argentina) and water *ad libitum.* Athymic nude mice of both sexes, 2–4 months old were obtained from the Comisión Nacional de Energía Atómica, Argentina and kept under relatively aseptic conditions. Animals were age and sex matched within each experiment. Care of animals was according to the policies of NIH Guide and Use of Laboratory Animals.

### Tumor

MC-C: Fibrosarcoma developing in a 5 month old BALB/c male, 3 months after the subcutaneous (s.c.) implantation of a methylcholanthrene pellet. It is a strongly immunogenic tumor with a tumor dose 50 (TD_50_ = the number of tumor cells able to grow in 50% of mice inoculated) of 5 × 10^4^ tumor cells (Franco et al. 1996). It can be maintained by serial s.c passages or biofrozen in liquid nitrogen until use. In this paper, MC-C tumor was used between s.c passages 10–15. Tumor volume was calculated according to the formula of Attia and Weiss: volume = 0.4 *ab^2^*, where *a* and *b* are the larger and smaller diameters respectively (Franco et al. 1996). Although different definitions of established tumors have been proposed (Yu et al. 2006), in this paper, we refer to “established tumor” as one clinically detectable, that is, evidenced by palpation and suitable for caliper measurement.

### Preparation of tumor lysate

Tumor tissue was removed and tumor cells were dispersed in phosphate buffered saline (PBS) to create a single-cell suspension. Cells were lysed by four to five freeze cycles (on liquid nitrogen) and thaw cycles (room temperature). Larger particles were removed by centrifugation (10 min, 1800 r.p.m.), supernatants were sonicated for 10 min in a Branson Digital Sonifier and then passed through a 0.2-μm filter, protein content determined and adjusted at 7.5 μg/ml and aliquots stored at −80 ºC until use.

### Isolation of bone marrow-derived dendritic cells

Femurs and tibiae of mice were removed and freed of muscles and tendons. The bones were placed in 70% ethanol for 2 min and subsequently washed in PBS. Both bone ends were cut off and the marrow was flushed out with RPMI 1640 medium (Gibco, Grand Island, NY). The red cells were lysed with ammonium chloride (0.45 M). The cells were centrifuged for 10 min at 1,500 rpm and 2 × 10^5^/ml cells were cultured in Petri dishes in 10 ml of RPMI1640 medium with 10% fetal bovine serum (FBS); 2 mM L-glutamine, 100 U ml^−1^ penicillin and 100 mg ml^−1^ streptomycin (complete medium) and supplemented with 5 × 10^−5^ M 2-mercaptoethanol and 15%–30% of mouse granulocyte-macrophage colony-stimulating factor (mGM-CSF)—containing supernatant from a J558 cell line stably transfected with mGM-CSF. On day 8, more than 95% of the cells were CD11c+ (immature dendritic cells).

### Vaccination strategy based in dendritic cells (DC) pulsed with tumor lysate

**Figure 1:**
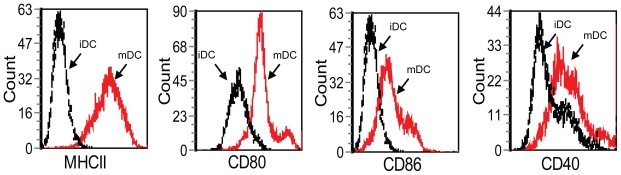
Representative experiment showing the expression of the molecular markers of maturation MHCII, CD80, CD86 and CD40 in dendritic cells (DC) pulsed with MC-C tumor lysate for two days in culture (mature DC or mDC), revealed with fluoresceinated specific antibodies. Controls were dendritic cells pulsed with none (immature DC or iDC).

**Figure 2:**
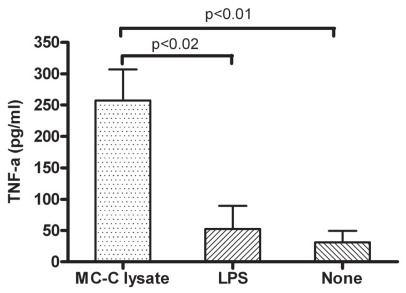
High titer of TNF-α in the supernatant of DC pulsed with MC-C tumor lysate for two days in culture. Supernatants of dendritic cells pulsed with LPS or none, served as control. The values represent the mean ± standard error of 4 experiments.

Bone marrow-derived day 8 DC (1 × 10^6^) were incubated in 1 ml of complete medium with 0.25 ml of MC-C tumor-lysate at 37 ºC. After two days in culture, a significant proportion (more than 75%) of mature DC (mDC) were evidenced by their molecular markers of maturation CD40, CD80, CD86 and MCH II (Fig. 1) and by the high titer of TNF-α displayed by supernatants of these cells as compared with that observed in untreated and LPS-treated DC that served as controls (Fig. 2). After washing, 3 × 10^5^ mDC were injected s.c. in the foot pad of recipient mice at selected times before or after tumor challenge. DC pulsed with LPS or not pulsed were injected as control.

### Determination of Tnf-α

Mouse TNF-α concentration was evaluated by using an ELISA kit from RD Systems, Minneapolis, MN, following manufacturer’s recommendations.

### Flow cytometry

Dendritic cells (1 × 10^6^ cells) were reacted with 100 ng of fluoresceinated anti-CD11c, anti-CD40, anti-CD80, anti-CD86 and anti-MHCII (Santa Cruz Biotechnology, Santa Cruz, CA). Irrelevant isotype-matched Abs were used as controls. Cells were reacted with the desired Ab for 30 min at 4 ºC and then washed in PBS with 1% FBS. Samples were analyzed for fluorescence using a Coulter Profile II flow cytometer (Palo Alto, CA).

### Statistical analysis

Student’s t-test and Logrank test were used. Values were expressed as mean ± standard error. Differences were considered significant whenever the p value was 0.05 or smaller.

## Results

### Preventive vaccination and therapeutic effects of vaccination against established tumors

BALB/c mice received a single dose of mature dendritic cells pulsed with tumor lysate (3 × 10^5^ mDC intra footpad), 10 days prior tumor inoculation (5 × 10^5^ MC-C tumor cells by the subcutaneous route) or at 5, 8, 12, 18, 25 and 35 days after tumor inoculation, when tumor volumes were 10 mm^3^, 50 mm^3^, 250 mm^3^, 600 mm^3^, 1000 mm^3^ and ≥2000 mm^3^ respectively. MC-C tumor growth was registered in these mDC treated mice as well as in euthymic mice receiving immature DC (iDC) or none, which served as controls. In some cases, tumor growth was also evaluated in nude mice.

**Figure 3:**
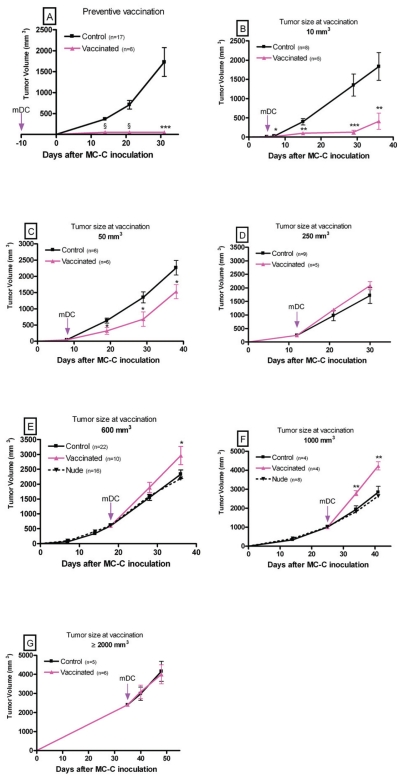
Growth of MC-C initiated with a s.c. inoculum of 5 × 10^5^ MC-C tumor cells in mice receiving a vaccine consisting in a single dose of 3 × 10^5^ mDC intra footpad, in a preventive mode, 10 days prior tumor inoculation (**A**) or in a therapeutic mode, at 5 (**B**), 8 (**C**), 12 (**D**), 18 (**E**), 25 (**F**) and 35 (**G**) days after tumor inoculation, when tumor volumes were 10 mm^3^, 50 mm^3^, 250 mm^3^, 600 mm^3^, 1000 mm^3^ and ≥2000 mm^3^, respectively. Growth of MC-C inoculated in mice receiving iDC or none served as controls. In E and F growth of MC-C tumor in nude mice was registered in addition to that in vaccinated and control euthymic mice.*p < 0.05; **p < 0.02; ***p < 0.01; §: p < 0.001.

**Figure 4:**
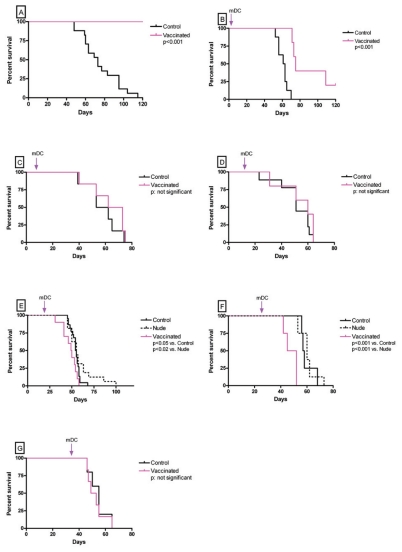
Survival curves of vaccinated and control mice from Figure 3A–G.

When mDC were inoculated prior to tumor inoculation (*preventive mode*), MC-C tumor growth was completely abolished (Fig. 3A) confirming previous reports showing that MC-C is a strongly immunogenic tumor (Franco et al. 1996; Chiarella et al. 2007 *a* and *b*). Survival of immunized and control mice are shown in Figure 4A.

On the other hand, when mDC were used in a *therapeutic mode*, the fate of MC-C tumor was dependent on its size at the time in which vaccination was carried out.

In effect, when mDC were inoculated into mice bearing incipient tumors (10 mm^3^ or 50 mm^3^), tumor growth was slowed down as compared with controls (Figs. 3B and C). This inhibitory effect was more striking in mice bearing 10 mm^3^-sized tumors than in those bearing 50 mm^3^-sized ones. This was reflected in the fact that only in the former, survival was significantly prolonged as compared with controls (see Figs. 4B and C).

When mDC were inoculated into mice bearing tumors measuring 250 mm^3^ or ≥2000 mm^3^, a null effect was detected, that is, tumor growth (Figs. 3D and G) and survival (Figs. 4D and G) were similar to those observed in controls.

Finally, when mDC were inoculated into mice bearing intermediate-size tumors (600 mm^3^ or 1000 mm^3^), a significant acceleration of tumor growth (Figs. 3E and F) and reduced survival (Figs. 4E and F) were evident, not only as compared with untreated euthymic BALB/c but also nude mice. It is worth noting that growth of MC-C tumor in nude mice was slightly faster than in euthymic BALB/c mice during the first two weeks after tumor inoculation, although afterwards, beyond 400–500 mm^3^ of tumor size, kinetics were identical in both types of mice.

#### Therapeutic effects of vaccination against residual tumor cells after debulking established tumors

In clinical settings, most immunotherapeutic schedules have been attempted after debulking the primary tumor by surgery, radiation or chemotherapy (Cranmer, 2004; Mocellin et al. 2004; Offringa, 2006; Morgan et al. 2006). The rationale for this practice is the assumption that the immune system can efficiently deal with a limited number of tumor cells, such as that present in a small tumor fragment or in an incipient tumor, but not with a large one, such as that present in a larger growing tumor. On this basis, tumor measuring about 600 mm^3^ or 2400 mm^3^ were partially excised leaving behind a small mass of 2 × 10^6^ MC-C residual tumor cells. The number of MC-C cells left behind was calculated indirectly in 12 mice which were similarly operated upon and sacrificed to count the remaining tumor cells. Reliability of this method (Ruggiero et al. 1985) was indicated by the fact that differences in tumor cell counting did not exceed 15% of the mean value (2 × 10^6^).

The fate of the MC-C tumor cell left at the operation site in otherwise untreated mice was compared with that of mice receiving mDC in the footpad, three days after surgery.

**Figure 5:**
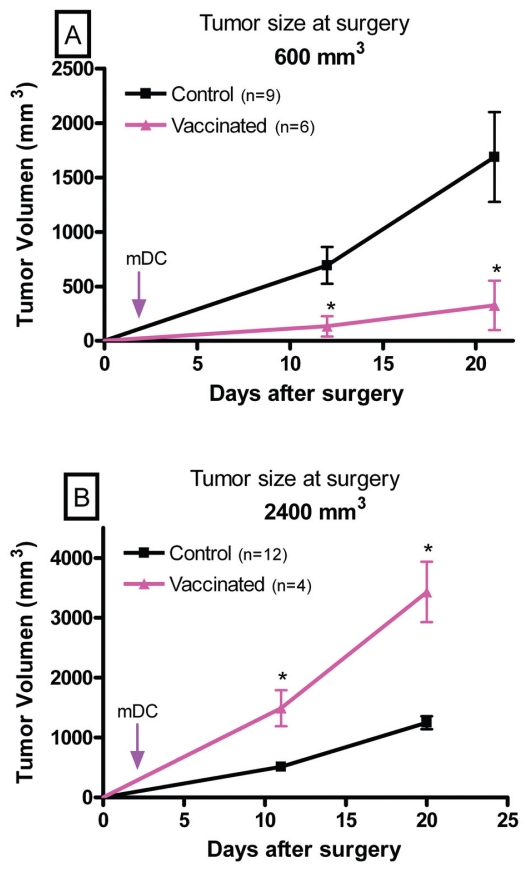
Growth of 2 × 10^6^ residual tumor cells left behind at the operation site after partially debulking a s.c. MC-C tumor measuring 600 mm^3^ (**A**) or 2400 mm^3^ (**B**) at the time of surgery, in mice receiving a vaccine of mDC 3 days after surgery (vaccinated) or in controls receiving none after surgery. *p < 0.05

**Figure 6:**
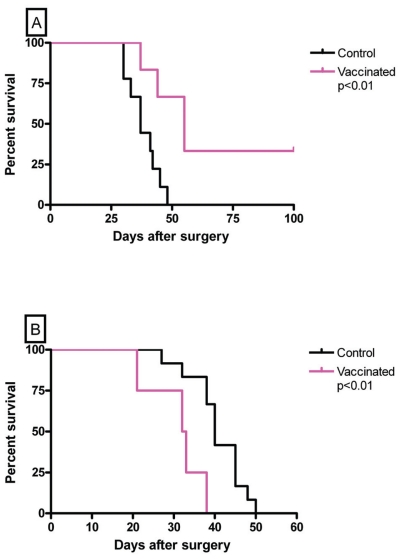
Survival curves of vaccinated and control mice from Figure 5A–B.

Results were strikingly different depending on the size of the tumor excised. In effect, when the size of the excised tumor was about 600 mm^3^, treatment with mDC significantly slowed down the growth of the residual tumor cells (Fig. 5A) and significantly prolonged the survival of the mDC-treated mice (Fig. 6A).

In contrast, when the size of the excised tumor was about 2400 mm^3^, treatment with mDC significantly enhanced tumor growth (Fig. 5B) and significantly reduced the survival of the mDC-treated mice (Fig. 6B).

## Discussion

Since the pioneer work of Prehn and Main (1957) demonstrating that *preventive vaccination* against some experimental murine tumors was feasible, there have been numerous attempts to treat human tumors using immunological schedules. However, the results have, in general, been disappointing (Finn 2003; Cranmer et al. 2004; Vieweq et al. 2007).

A weak (or a lack of) antigenicity of human cancers as well as strategies used by tumors to acquire resistance to apoptosis have been claimed as putative explanations for these failures.

However, it is clear from previous observations and from the results obtained herein, that anti-tumor vaccines are ineffective to treat even strongly immunogenic tumors.

That is, the same immunological procedures which successfully prevent the growth of implants of tumor cells are, on the contrary, largely inefficient to eradicate, inhibit or even slow the growth of the same tumor cells when they are growing as a tumor larger than a minimal critical size (Chiarella, 2007 *a* and *b*; North, 1984; Mayordomo et al. 1995; Heckelsmiller et al. 2002).

The simplest interpretation of this fact would be that the number of immune-reactants that are generated by a given vaccine (herein, a single dose of a dendritic cell-based vaccine) is enough to efficiently deal with a small number of tumor cells such as that present in a small tumor inoculum or in an incipient tumor but not in a larger growing tumor.

However, this interpretation presents two main difficulties:

1. Tumor-inhibitory capacity of the dendritic cell-vaccine utilized herein did not seem to be a linear function of the ratio *number of immune-reactants/number of tumor cells*. In effect, the inhibitory capacity of the vaccine was very strong when it faced a small implant of 5 × 10^5^ tumor cells; thereafter, its inhibitory effect was progressively lower as the number of tumor cells was increasingly larger (incipient tumors measuring 10 and 50 mm^3^) until its effect disappeared when the tumor size was about 250 mm^3^. However, when the vaccine faced intermediate-size tumors measuring 600 and 1000 mm^3^, its effect was not null but stimulatory of tumor growth and later, when tumors were large (≥2000 mm^3^) its effect was null again, that is neither inhibitory nor stimulatory of tumor growth. Moreover, tumor growth in vaccinated-BALB/c mice bearing intermediate-size tumors was faster not only than in non-vaccinated BALB/c mice but also than in nude mice. This means that, in these cases, vaccination rendered worse consequences for the organism not only than mere no-treatment but also than a severe immunodeficiency such as that present in nude mice.
2. When partial tumor excision was carried out, the fate of the mass of tumor cells left behind at the operation site was strikingly different upon vaccination treatment, depending on the tumor size at the time of surgery. In effect, when an intermediate-size tumor (600 mm^3^) was partially excised, the vaccination treatment significantly inhibited the growth of the residual tumor cells. In contrast, when a large tumor ( 2400 mm^3^) was partially excised, the same number of residual tumor cells grew significantly faster in mice receiving the vaccine than in untreated mice.

We suspect that the sometimes neglected concepts of “immunostimulation” and “immunological eclipse” may be important to understand the problems of tumor immunotherapy.

The “immunostimulatory theory” proposed by Prehn states that the immune reaction may be either stimulatory or inhibitory to tumor growth depending upon the local ratio of immune reactants to tumor cells with *high* ratios rendering tumor inhibition, *intermediate* and *very low* ratios rendering null effects and *low* ratios (between intermediate and very low ones) rendering tumor stimulation (Prehn, 1972; Prehn, 2006; Prehn, 2007).

On the other hand, the “immunological eclipse” observed in hosts bearing tumors exceeding a minimal critical size, is characterized not only by the absence of anti-tumor immune response but by the emergence of a suppressor mechanism that sterilizes any attempt of active and passive immunotherapy (Chiarella, 2007 *b*; North, 1984). Different mechanisms have been reported to be associated etiologically with the immunological eclipse. Some of these impediments might be tumor cell-associated, including the loss of class I MHC expression, tumor-expression of the vascular cell adhesion molecule-1 (VCAM-1), enhanced expression by tumor cells and by the surrounding stromal cells of the tolerogenic enzyme indoleamine 2,3-dioxygenase (IDO) or the production by tumor cells of factors such as IL-10, TGF-β or galectin-1 that could inhibit effective immune responses (Lin et al. 2007; Rubinstein et al. 2004; Waldman, 2003; Zheng et al. 2006). Other impediments have been associated with a series of negative immunoregulatory mechanisms including the anomalous expression of the negative co-stimulatory molecule CTLA-4 in cytotoxic T lymphocytes, the increased expression of CD4+CD25+ T reg or CD4+ NKT cells, the emergence of a systemic inflammatory condition, etc. (Waldman, 2003; Chiarella et al. 2007 b).

In our model, the immunological eclipse is observed when the tumor grows beyond 500 mm^3^; it is partially reversible when intermediate-size tumors ranging 500–1500 mm^3^ are surgically excised but it is not reversible when larger tumors are excised (unpublished observations).

To account for the results obtained herein, a numeric example integrating the above mentioned concepts may be illustrative. This example is based on the following considerations:

*First*: Since, in our experience, a tumor fragment measuring 1 mm^3^ has 4 × 10^5^ tumor cells, approximately, tumors measuring 10, 50, 250, 600, 1000 and ≥2000 mm^3^, will have 4, 20, 100, 240, 400 and ≥800 × 10^6^ tumor cells, respectively.

*Second:* The number of immune-reactants produced by the vaccine inoculated into immunocompetent mice (i.e. normal mice or mice bearing tumors smaller than 500 mm^3^) must be high (for example 100 × 10^6^); in contrast, the number of immune-reactants must be very low (for example 0.5 × 10^6^) when the vaccine is inoculated into “eclipsed” mice (i.e. mice bearing tumors larger than 500 mm^3^) as a resultant of the number theoretically produced by the vaccine counteracted by the suppressor elements. When intermediate-size tumors are excised, partial reversion of the immunological eclipse must, upon vaccination, be evidenced by an intermediate number of immune-reactants (for example, 20 × 10^6^); on the other hand, when a large tumor is excised, no reversion of the immunological eclipse is observed and in consequence, the number of immune-reactants must, upon vaccination, remain very low (0.5 × 10^6^). The number of anti-tumor immune-reactants in tumor-bearing nude mice must be near zero because even xenogeneic tumors can grow in them. Similarly, in non-vaccinated tumor-bearing BALB/c mice, the number of immune-reactants, must also be near zero, especially in “eclipsed” mice, because in both nude and eclipsed BALB/c mice, tumor growth proved to be identical.

On this basis, the ratio: *number of immune-reactants/number of target tumor cells,* can be calculated in the different cases presented herein.

In effect, when the vaccine was used in a *preventive* manner (PM) or inoculated *therapeutically* in mice bearing tumors measuring 10 (T10), 50 (T50), 250 (T250), 600 (T600), 1000 (T1000) and ≥2000 (T ≥ 2000) mm^3^, the ratios must have been, respectively:

$$\begin{array}{l} \text{PM}=100\times 10^{6}/0.5\times 10^{6}=200;\text{T}10=100\times \\ 10^{6}/4\times 10^{6}=25;\text{T}50=100\times 10^{6}/20\times 10^{6}=5;\\ \text{T}250=100\times 10^{6}/100\times 10^{6}=1;\text{T}600=0.5\times \\ 10^{6}/240\times 10^{6}=0.00208;\text{T}1000=0.5\times 10^{6}/400\times \\ 10^{6}=0.00125;\text{T}\geq 2000=0.5\times 10^{6}/\geq 800=\\ \leq 0.000625. \end{array}$$

Similarly, when the vaccine was inoculated *therapeutically* after debulking a tumor measuring 600 mm^3^ (DT600) or ≈ 2400 mm^3^ (DT2400), the ratios must have been, respectively:

$$\begin{array}{l} \text{DT}600=20\times 10^{6}/2\times 10^{6}=10;\text{DT}2400=\\ 0.5\times 10^{6}/2\times 10^{6}=0.25 \end{array}$$

In tumor bearing nude (N) and in non-vaccinated tumor bearing “eclipsed” BALB/c (B) mice, the ratios would be near 0 independently of the number of target tumor cells, because the numerator would be near zero in all cases.

This example, although oversimplified and theoretical (especially concerning the real number of immune-reactants cells in each experimental setting), might account for the results described in this paper by assuming, on the basis of the immunostimulatory theory, that ratios higher than 1 (PM, T10, T50 and DT600), render tumor inhibition; ratios near 1 (T250) and lower than 0.001 (T ≥ 2000, N and B) render null effects and ratios lower than 1 but higher than 0.001 (T600, T1000 and DT2400) render tumor stimulation.

The controversial observations that at a tumor size of 600 mm^3^, a vaccination with mature DC showed an acceleration of tumor growth with the opposite effect after excision of the tumor and that at a tumor size of 2400 mm^3^ a null effect on tumor growth was observed upon mature DC vaccination with an enhancement of tumor growth by mature DC after tumor excision, merit a more extensive comment. According to the immunostimulatory theory, the local ratio between immune-reactants and target tumor cells, will define the outcome of the tumor, with *high* ratios rendering tumor inhibition, *intermediate* and *very low* ratios rendering null effects, and *low* ratios rendering tumor stimulation. In the case of mice bearing tumors measuring 600 mm^3^ receiving the DC vaccine, this ratio must be *low* (in our example = 0.00208), taking into account, firstly, that the immunological eclipse (displayed when MC-C tumor is larger than 500 mm^3^) precludes the existence of a high number of anti-tumor immune cells upon vaccination and, secondly, that the tumor exhibits a relatively high number of tumor cells. This *low* ratio would generate tumor immunostimulation with a consequent enhanced tumor growth. Conversely, after debulking the tumor, the number of tumor cells is sharply reduced and immunological competence is partially restored because, in our model, the immunological eclipse is partially reversible when tumors ranging 500–1500 mm^3^ are excised. In consequence, the ratio between number of immune-reactants and number of tumor cells after vaccination will become relatively *high* (in our example = 10) which can generate inhibition of tumor growth. On the other hand, in the case of mice bearing tumors measuring 2400 mm^3^ receiving the DC vaccine, the ratio must be *very low* (in our example = 0.000625) taking into account, firstly, that the immunological eclipse precludes the existence of a high number of anti-tumor immune cells upon vaccination, and secondly, that the tumor exhibits a very high number of tumor cells. This *very low* ratio would generate a null effect on tumor growth. Conversely, after debulking the tumor, the ratio will become *low* after vaccination (in our example = 0.25) because, even though the number of tumor cells is sharply reduced, number of immune-reactants remains very low because immunological competence is not restored after debulking a tumor larger than 1500 mm^3^. Therefore, this *low* ratio would generate a stimulatory effect on tumor growth.

In conclusion, data presented herein suggest that immunotherapy against established tumors or against residual tumor cells after debulking the primary tumor, can produce inhibitory, null or stimulatory effects on tumor growth. This would depend upon where the system stands on the immune response curve relating the magnitude of the immune response to the quantity of target tumor cells. This in turn would depend upon the antigenic profile of the tumor, the immunogenic strength of the vaccine used, and the immunological state of the host (immune-competence or eclipse).

*In vitro* and *in vivo* studies aimed to determine the magnitude of these variables, seem to be necessary as a pre-condition for designing rational immunotherapeutic approaches to cancer that could optimize the effects of an active or passive immunological treatment converting a null or stimulatory ratio between immune-reactants and target cells to an inhibitory one, for example by eliminating of ameliorating the immunological eclipse or by altering the immunogenic strength of the treatment.

In contrast, if these studies are neglected, the worst thing that an immunotherapist could face is not merely a null effect but enhancement of tumor growth and earlier death of the patients.
